# SOCIAL DETERMINANTS AND HEALTH EQUITY AMONG NYC OLDER ADULTS

**DOI:** 10.1093/geroni/igad104.2352

**Published:** 2023-12-21

**Authors:** Duy Nguyen, Carson De Fries, Ethan Siu Leung Cheung

**Affiliations:** Sacred Heart University, Fairfield, Connecticut, United States; University of Denver, Denver, Colorado, United States; University of Utah, Salt Lake City, Utah, United States

## Abstract

Of the vast opportunities in New York City, older adults from racial/ethnic minority groups face the challenges of social determinants on their health. This study applies the WHO’s Commission on Social Determinants of Health model to examine health inequities among older adults in New York City. A deeper understanding of interrelationship between structural and intermediary determinants is crucial to promoting successful aging in an urban environment. Data were extracted from the NYC Community Health Survey to focus on older adults, aged 65 and over (N=1,921). Weighted, limited information regression models were used to test the contributions of structural and intermediary determinants on self-reported daily activity difficulties and self-reported health. The sample comprised of 46% Non-Hispanic White older adults, 20% Black/African American, and another 20% Hispanic. One third of the sample endorsed being in poor health. One in 5 older adults reported having a daily activity difficulty. Multivariate analyses showed Black older adults reported more difficulties with daily activities compared to non-Hispanic Whites. Meanwhile, Hispanic older adults reported being in poor health less frequently than non-Hispanic Whites. Further limited information modeling shows the centrality of daily activity limitations on poor health such that the indicator partially mediates the relationship between race/ethnicity and poor health. Overall, the results reveal a fuller picture of the impact of social determinants on health equity among older adults in New York City. Future research needs to specify the sociocultural and economic dimensions of health equity to provide empirical results that can inform practice and policy.

